# Corneal Neurotization—Indications, Surgical Techniques and Outcomes

**DOI:** 10.3390/jcm12062214

**Published:** 2023-03-13

**Authors:** Diana Carmen Dragnea, Iva Krolo, Carina Koppen, Callum Faris, Bert Van den Bogerd, Sorcha Ní Dhubhghaill

**Affiliations:** 1 Department of Ophthalmology, Antwerp University Hospital, 2650 Edegem, Belgium; 2 Department of Medicine, University of Antwerp, 2610 Wilrijk, Belgium; 3 Department of Otolaryngology, Antwerp University Hospital, 2650 Edegem, Belgium; 4 Antwerp Research Group for Ocular Science (ARGOS), Department of Translational Neurosciences, University of Antwerp, 2610 Wilrijk, Belgium

**Keywords:** corneal neurotization, neurotrophic keratopathy, anesthetic cornea, corneal sensation, corneal nerves, nerve coaptation, nerve graft

## Abstract

Corneal neurotization is a promising surgical approach for the treatment of moderate to severe neurotrophic keratopathy. This technique aims to restore corneal sensation by transferring healthy nerves, either directly or via a conduit, to the anesthetic cornea. This review provides a report on the current state of development, evidence, and experience in the field. We summarize the data available from clinical reports and case series, placing an emphasis on the diversity of the surgical techniques reported. While these data are encouraging, they also highlight the need for a consensus in reporting outcomes and highlight how the next step will involve validating putative outcome parameters when researching and reporting corneal neurotization surgery.

## 1. Introduction

**Figure 1:**
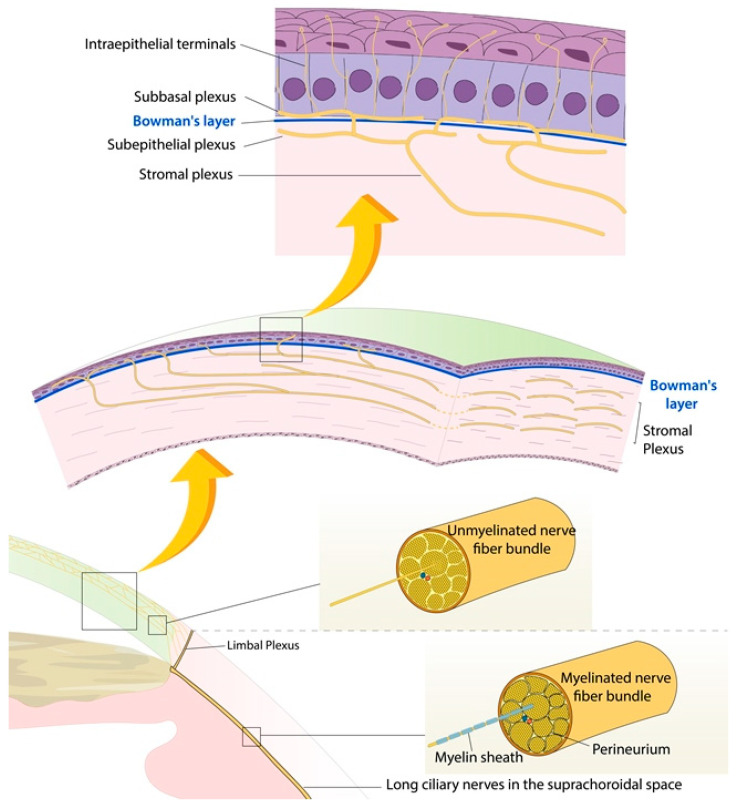
Corneal nerves anatomy.

The human cornea is innervated by a very dense network of fibers, comprising a limbal nerve plexus, a stromal plexus, a subepithelial plexus beneath Bowman’s membrane, a sub-basal plexus above Bowman’s membrane, and the intraepithelial terminals (Figure 1) [1,2,3,4,5]. Most of these fibres are sensory, though there is some contribution for the autonomic nervous system. The sensory nerves are predominantly derived from the ophthalmic branch of the trigeminal nerve, but some contributory fibres of the maxillary branch of the trigeminal nerve have been described [6]. Upon exiting the trigeminal ganglion, approximately 40 thick nerve bundles travel suprachoroidally and converge on the cornea, approximately 1 mm from the limbus, forming the limbal plexus [6]. As the nerves leave the plexus and enter the cornea, they shed their myelin sheaths and perineurium. The absence of myelin allows the nerves to remain transparent as they traverse the cornea. In some normal corneas, however, the nerves can remain prominent and can be visible on clinical slit-lamp examination, though this can also be associated with certain conditions such as multiple endocrine neoplasia [7] and keratoconus [8].

The density of free nerve endings in the suprabasal epithelial plexus is one of the highest in the human body with approximately 606 terminals per square millimeter [5]. These terminals have wide, overlapping receptive fields, with a higher density centrally than peripherally, which may reflect the importance of protecting the central cornea. While transmission of pain sensations is a key function of these nerves, their importance is not limited to that. Approximately 70% of the receptors are polymodal, serving heat, inflammatory, and chemical irritant sensations; 20% are mechanoreceptors; and the final 10% relay the sensation of cold [9].

These nerve endings serve more than just sensations as they also provide the inputs to the afferent limbs of both the blink reflex and the lacrimal functional unit, the latter being responsible for the secretion of the tear film [1,2]. The presence of healthy nerves also provides crucial trophic support to the corneal epithelial cells. Neurotrophic factors known to play a role in epithelial integrity include Nerve Growth Factor (NGF), Brain-Derived Neurotrophic Factor (BDNF), Glial Derived Neurotrophic Factor Neurotrophin 3, Semaphorin 3A, 3F and 7A, substance P, and Neuropeptide Y (NPY), though others have been described [10,11].

**Figure 2:**
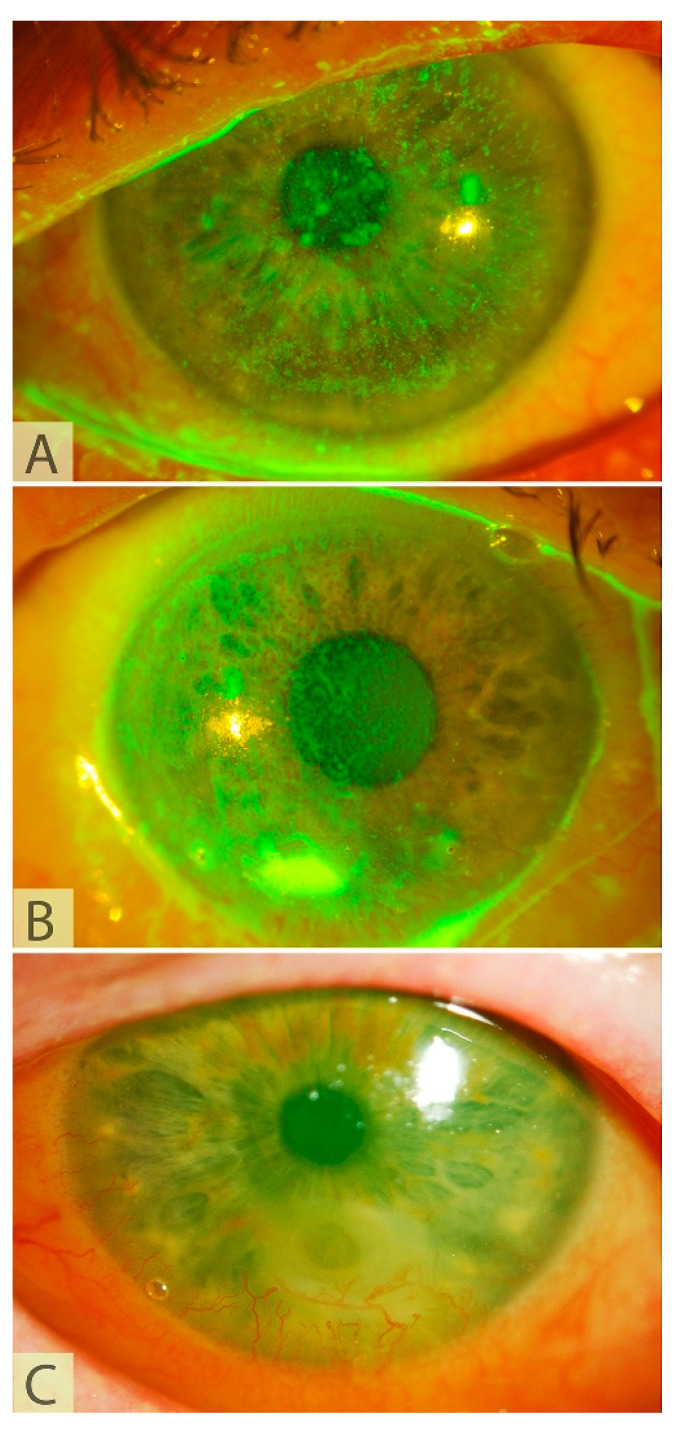
Representative pictures of our patients showing different Mackie stages of neurotrophic keratopathy: (**A**) Stage I: punctate keratopathy without epithelial defect; (**B**) Stage II: persistent epithelial defect without stromal involvement; (**C**) Stage III: stromal involvement and Descemetocele.

Loss of corneal sensation can result from a wide range of corneal insults, including congenital, systemic, infectious, or toxic causes, and external or surgical trauma at any level of the innervation, from the brain to the corneal nerve endings [11]. The most common causes include viral herpetic infection, chemical burns, and surgical trauma [12]. The loss of nerve function can impair blinking, reduce tear production, and can disturb epithelial homeostasis, resulting in a cascade of pathological corneal changes known as neurotrophic keratopathy (NK). Clinically, NK can be suspected based on the history and the absence of pain, despite manifest corneal epithelial changes. NK may be graded into one of three stages, based on the Mackie classification (Figure 2) [13]. Stage 1 is a milder presentation with an intact, albeit irregular, corneal epithelium, with punctate keratopathy that may be accompanied by stromal scarring. Stage 2 is characterized by persistent epithelial defects with a thickened “rolled up” epithelium at the margin. In stage 3, the persistent epithelial defect renders the stromal tissue vulnerable to melting and perforation. Both corneal melting and perforation can be expedited by inappropriate use of corticosteroid or non-corticosteroid anti-inflammatory medications or a secondary infection.

While the pathophysiology of neurotrophic keratitis may be complex, the medical management is quite limited and focused on maintaining an adequate corneal tear film, though this may not improve vision [14]. Artificial tears and thicker lubricants, bandage contact lenses, punctal occlusion, protective glasses, and shields can be used, particularly in the presence of lagophthalmos, to manage milder grades of NK [11].

More severe cases may benefit from the use of topical recombinant NGF or of more cost-effective treatment options such as oral nicergoline and topical insulin [15,16,17,18]. The use of recombinant NGF is currently limited by two issues: firstly, the cost and, secondly, that it appears to be most effective when there is residual conjunctival sensation. This may be related to residual nerve presence in the ocular surface. NGF can likely attract growth to the cornea from the conjunctiva but in the total absence of ocular surface nerves it is unlikely that NGF can create entire nerves de novo. Treatment with oral nicergoline was shown to help patients with NK in whom lubricants and contact lenses did not bring clinical improvement [17]. Recently, the combination of oral nicergoline, autologous serum, and bandage contact lenses for the management of stage III NK showed complete epithelial healing in a small cohort of eight patients [19].

Surgical options are typically reserved for recalcitrant cases (i.e., severe grade 2 or 3) where medical/non-surgical interventions cannot prevent the impending risk of melting or perforation. The standard surgical management of NK includes suturing of amniotic membranes, conjunctival flaps, botulinum toxin ptosis, tarsorrhaphy, as well as the recently described pillar tarsoconjunctival flap [20], all of which manage the consequences of the anesthesia rather than addressing the underlying deficit.

Corneal neurotization is a promising surgical approach for the treatment of moderate to severe NK. This technique aims to restore corneal sensation by transferring healthy nerves, either directly or via a conduit, to the anesthetic cornea. This review provides a report on the current state of development, evidence, and experience in the field.

## 2. Method of Literature Review

A PubMed literature search was performed to identify available studies regarding corneal neurotization until December 2022. There were no limits placed on study publication date, publication status, minimum follow-up time, or design. Relevant studies were identified by utilizing the following search terms: corneal neurotisation/neurotization, corneal nerves, and neurotrophic keratopathy. The articles were reviewed for their title, abstract, and language (English, French, German, Italian, and Russian were included). The non-English articles were translated. In addition, the references of the articles were scanned to identify additional resources. At this point, we included 52 articles for corneal neurotisation/neurotization, 9 articles for corneal nerves and 11 articles for neurotrophic keratopathy. In case of incomplete or unclear information, direct communication with the authors was enacted. After this, a new PubMed search about cadaveric studies (Chapter 5) was performed utilizing the following terms: sural nerve, supraorbital nerve, supratrochlear nerve, infraorbital nerve, great auricular nerve, sural nerve harvest; six more articles were found. Finally, for Chapter 6, we included 19 additional articles.

Outside of PubMed database, we found relevant information in one conference presentation from Dr. Cristina Menicacci [21]. Direct communication with Prof. Dr. med. M. Samii helped us include older but valuable resources [22,23].

## 3. Indications for Corneal Neurotization

Corneal neurotization aims to protect the corneal integrity by restoring the epithelial sensitivity and its trophic support, thereby promoting epithelial closure and thus inhibiting stromal lysis. The technique has been described in both adults and children [24]. The clinical entities where corneal neurotization has been used are as broad as all the ocular and systemic conditions than can cause severe NK (Table 1). The most frequent indication for neurotization is surgical nerve damage (around 60 cases described in the literature to date) which was most acquired after intracranial surgery [25]. The second most frequent indication found in the literature was in the treatment of neuropathy associated with Herpes Simplex Virus (HSV) or Varicella Zoster Virus (VZV). After these two main indications, congenital nerve dysfunction, trauma, and long ciliary nerve damage after vitreoretinal surgery account for the majority of the other cases reported. Single cases for other indications, such as diabetic neuropathic keratopathy, have also been described.

## 4. Corneal Neurotization—Surgical Techniques

Corneal neurotization can be performed either by directly transferring a nerve from its original position to supply the cornea (direct corneal neurotization, DCN) or by connecting a nerve to the cornea via nerve graft conduit (indirect corneal neurotization, ICN) [26,27,28,29,30,31,32,33,34,35]. Direct neurotization could perhaps achieve a faster clinical response, while indirect neurotization requires new axons to grow down the length of the nerve graft to the cornea and can take approximately 6 months.

### 4.1. The History of Corneal Neurotization

**Figure 3:**
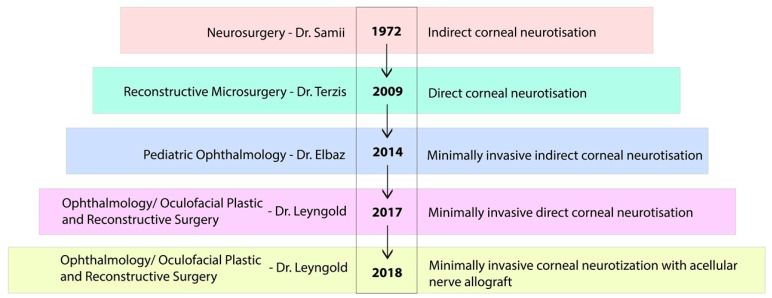
The history of corneal neurotization.

The history of corneal neurotization is punctuated by several landmark studies (Figure 3). The earliest approach was published in 1972 in the field of neurosurgery [22]. The first case ever performed was in 1969 on a 12-year-old child, after a severe trauma. The patient had lost vision in the left eye due to optic nerve trauma and had a major lesion of the trigeminal nerve on the right side (correspondence with the author). The right eye subsequently developed a neurotrophic ulcer, with significant loss of vision in his only functional eye. After two unsuccessful corneal transplantations, the procedure for restoration of corneal sensation was performed. An anastomosis between the dysfunctional ophthalmic nerve and the major occipital nerve with means of a sural nerve graft was performed in this case and for two other patients with neurotrophic keratitis (“ceratitis neuroparalytica”) [23]. The authors described an incomplete recovery of sensation, and all patients showed improvement of the cornea. While this complex approach of ICN through an osteoplastic frontal craniotomy was not widely adopted, it was the first proof of concept that neurotization of the cornea could work. There was little further development in the field for almost 40 years until the re-emergence of the concept in 2009 [36].

### 4.2. Direct Corneal Neurotization

**Figure 4:**
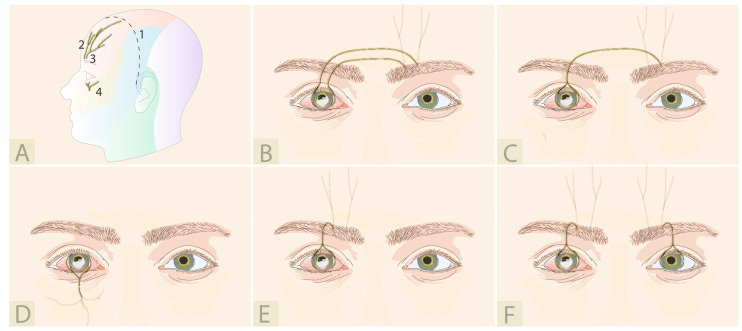
Direct corneal neurotization: (**A**) Representation of (1) coronal incision, (2) supratrochlear nerve, (3) supraorbital nerve, and (4) infraorbital nerve; (**B**) Contralateral supratrochlear and supraorbital [36,37,38,39]; (**C**) Contralateral supraorbital [27,41,42]; (**D**) Ipsilateral infraorbital [43]; (**E**) Ipsilateral supraorbital [42,44,45]; (**F**) Bilateral ipsilateral supraorbital [42].

All described variations of DCN are illustrated in Figure 4. In 2009, Terzis et al. described the first cases of a novel corneal neurotization technique [36,37]. This newer approach was considerably less invasive and required a bicoronal incision, rather than a frontal craniotomy. In this landmark paper, the healthy supraorbital and supratrochlear nerves, contralateral to the affected cornea, were dissected, mobilized, and tunneled over the nasal bridge to the upper eyelid crease. The nerve fascicle endings were then brought through the prepared sub-Tenon’s space and sutured with a 10-0 nylon to the perilimbal region. In 2014, Allevi et al. reported one case using a similar neurotization approach where a penetrating keratoplasty could be performed 6 months later [38]. This patient ultimately achieved 0.7 (decimal Snellen) best corrected visual acuity and this group has continued to publish in the field [38,39]. When Ting et al. performed this technique on two patients, the first was successful while the second, despite an early improvement, eventually opted for an evisceration after 2 years due to persistent severe eye pain which was not present prior to neurotization [40].

Since 2009, many modifications of the original technique have been described; the surgery can be performed through a smaller hemicoronal incision in cases in which the ipsilateral supraorbital nerve can be used (e.g., long ciliary nerve damage after retinal detachment surgery), thereby reducing the morbidity associated with the surgery [44]. Lueke at al. published a case where corneal neurotization was performed after a horseshoe keratoplasty using the contralateral supraorbital nerve and a hemicoronal access [27]. They created four intrastromal pockets, where the nerve fascicles endings were placed. Gennaro et al. described a case of direct corneal neurotization where the coronal incision is avoided by using an intact ipsilateral infraorbital nerve for reinnervation through a subpalpebral incision [43]. This is also the first report in which the second division (V2) of the trigeminal nerve was used for direct corneal neurotization.

### 4.3. Minimally Invasive Direct Corneal Neurotization

Despite surgical refinement, the bicoronal or hemicoronal approaches were still associated with extended operative time, visible scars, hematoma, and alopecia in hair-bearing skin, so a minimally invasive direct corneal neurotization (MIDCN) was introduced in 2017 [41]. Leyngold et al. described the first case using a technique of harvesting the contralateral supraorbital nerve under endoscopic guidance, avoiding a coronal incision and thus the associated complications [46]. The patient presented with NK following herpes zoster infection and showed an improvement of corneal sensation 3 months post-operatively. Four other cases were described by the same group using ipsilateral supraorbital nerve transfer under either direct visualization alone or using a combination of endoscopic and direct visualization [42]. Recently, Lin et al. described the largest series of direct ipsilateral supratrochlear nerve transfer in herpetic neuropathic keratopathy using a very small incision. Nine eyes could be directly neurotized using a 3-cm supra-eyebrow incision because of the proximity of the transferred nerve [45].

### 4.4. Indirect Corneal Neurotization

**Figure 5:**
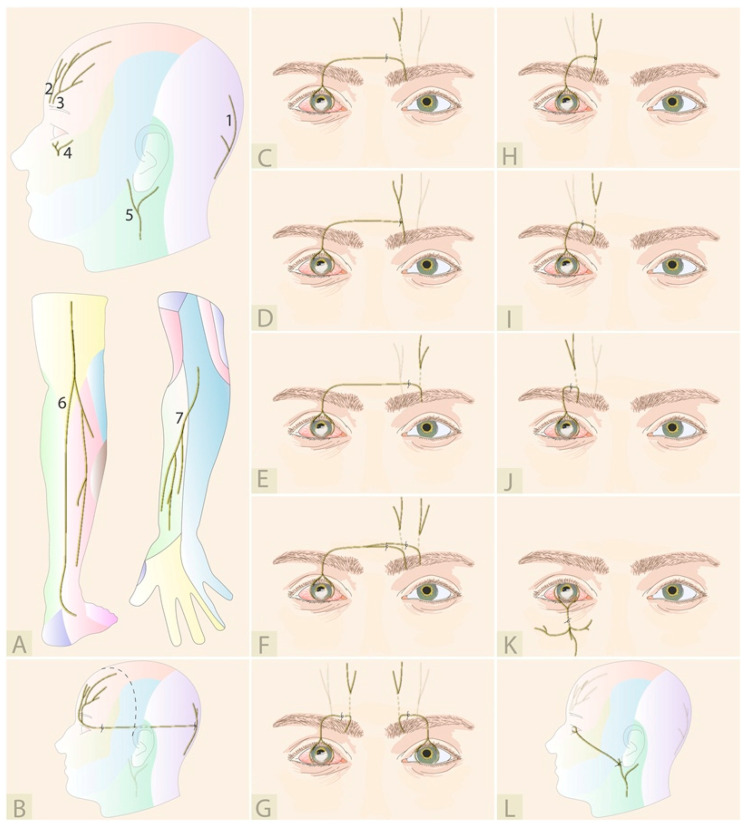
Indirect corneal neurotization using autograft: (**A**) Representation of (1) major occipital nerve, (2) supratrochlear (ST) nerve, (3) supraorbital (SO) nerve, (4) infraorbital (IO) nerve, (5) great auricular nerve (GAN), (6) sural nerve, (7) lateral antebrachial cutaneous (LAC) nerve; (**B**) Ophthalmic-sural-major occipital anastomosis [22]; (**C**) End-to-end sural [28,49,50,51,52,53,54] or GAN to contralateral ST nerve anastomosis [55]; (**D**) End-to-side sural to contralateral ST anastomosis [49,51,56]; (**E**) End-to-end sural [28,47,50,55,57,58] or LAC to contralateral SO nerve [59] anastomosis; (**F**) End-to-end sural to contralateral ST and SO [47,48]; (**G**) Bilateral end-to-end sural to ipsilateral ST [56]; (**H**) End-to-side sural to ipsilateral ST [47]; (**I**) End-to-end sural to ipsilateral ST [50,60]; (**J**) End-to-end sural to ipsilateral SO [47]; (**K**) End-to-end sural to ipsilateral IO [50,51]; (**L**) End-to side sural to GAN [61,62], bilateral case report [63].

**Figure 6:**
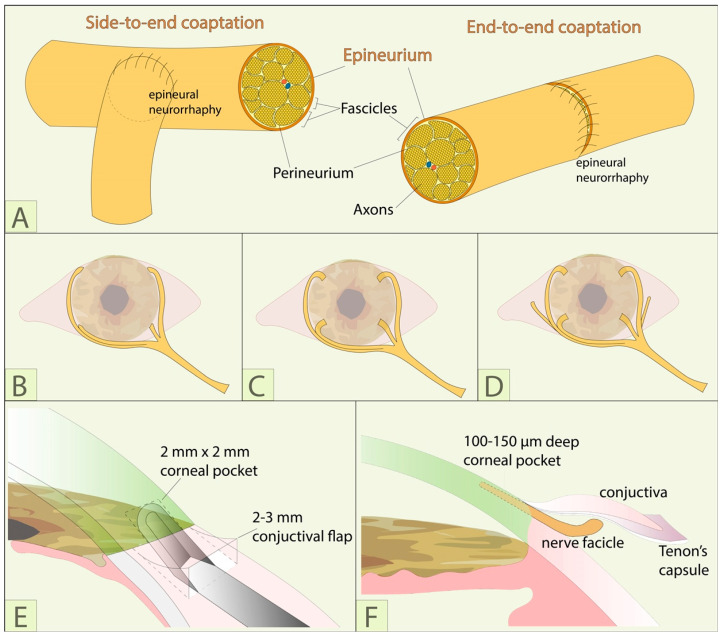
Surgical techniques of fascicle fixation: (**A**) Side-to-end and end-to-end epineural neurorrhaphy. Techniques of fascicle fixation in: (**B**) The sub-Tenon perilimbal space; (**C**) Sclero-corneal tunnels; (**D**) Sclero-corneal tunnels and perilimbal sub-Tenon’ space; (**E**,**F**) Close up of sclero-corneal tunnel preparation.

Some neurotization techniques use a nerve graft, like the sural graft originally used by Samii, to connect the source of innervation to the cornea (Figure 5). This use of a conduit nerve to relay the axons from source to the eye is known as indirect corneal neurotization. Indirect techniques require micro neurovascular surgical experience because these nerves must be surgically connected or “coapted”. Coaptations in corneal neurotization can be performed in either a linear “end-to-end” or branching “side-to-end” fashion (Figure 6A). The benefit of the “end-to-end” coaptation is that it maximizes the axonal load for neurotization but comes at the expense of the original dermatome supplied by the nerve. The “side-to-end” coaptation diverts fewer axons to the cornea but is associated with less loss of sensation in the area of the coapted nerve. While indirect techniques can reduce incision size and surgical time, the coaptation (or epineural neurorrhaphy) must be performed with the utmost care to avoid excessive tension on the nerve, fascicular damage, and problematic neuroma formation. Some authors choose a dual nerve-grafting approach using simultaneous sural nerve grafts from both the supratrochlear and supraorbital nerves to the affected contralateral cornea (Figure 5F) [47,48]. Some surgeons also recommend augmenting the coaptation by wrapping it with an amniotic membrane, placing the basement membrane down, which might support nerve growth through the connection [28].

#### 4.4.1. Clinical Reports and Case Series

In 2014, Elbaz et al. reported the first application of this indirect approach using the sural nerve, which was considerably less invasive than the older approach of the 1970s [56]. The surgery, called minimally invasive corneal neurotization (MICN), requires harvesting 10–15 cm of the sural nerve (median cutaneous branch), which could be performed during the same procedure. Briefly, the ipsilateral donor sensory supratrochlear nerve was accessed by a medial upper eyelid incision in a manner similar to the DCN technique. In these cases, the authors preferred end-to-side coaptation with the supratrochlear nerve to preserve some forehead sensation, but in one case an end-to-end coaptation was used due to a large size difference between the nerves. Finally, the graft nerve fascicles were tunneled into the cornea [49,56,60] or fixated to the sclera [58]. The outcomes of MICN are encouraging, showing improvement of corneal sensation and visual acuity [64]. An end-to-end sural nerve to contralateral supratrochlear coaptation has also been described in a case of a 2 year-old child with NK from left trigeminal nerve hypoplasia, which resulted in corneal improvement after only 8 weeks [54]. The beneficial effect of neurotization appears to persist, as long-term results of a larger MICN cohort (19 eyes of 16 patients) show improved corneal sensation and preservation of the ocular surface (19 eyes of 16 patients) [50].

Fung et al. subsequently described a modification where, in case of congenital nerve dysfunction, ICN was performed [51]. After discovering intraoperatively that the contralateral supraorbital and supratrochlear nerves were absent, the ipsilateral infraorbital nerve was used for coaptation to the sural nerve. In a second case, the same group described a successful deep anterior lamellar keratoplasty (DALK) 2.5 years after corneal neurotization, using a sural nerve graft coapted to the contralateral supratrochlear nerve. Weis et al. reported a prospective case series of patients who underwent successful corneal neurotization using a sural nerve graft [47]. All six patients had no complications and showed improvement in sensation, while five showed improved visual acuity with one patient awaiting corneal transplantation.

While most authors use supratrochlear and supraorbital nerves for neurotization, some authors chose the ipsilateral great auricular nerve (GAN) as the sensory source while keeping the sural nerve as an interponate [61,62,63]. The main arguments for using the GAN are that it contains a large number of sensory axons and that the loss of sensation is confined to a small part of the earlobe. The GAN had been used previously for neurotization, though as a graft interponate connecting the contralateral supratrochlear nerve to the cornea rather than providing the sensory axons themselves [65]. The rationale for using the GAN as a graft is that it would avoid the second operating field required for sural nerve harvesting and that it is a finer nerve, smaller than the sural and only slightly thicker than the supratrochlear. Recently, Bourcier et al. published a novel technique where they use the lateral antebrachial cutaneous nerve (LABCN) rather than the sural nerve, as the autologous graft for mini-invasive corneal neurotization (MICORNE) [59]. Using the LABCN for the neurotization resulted in excellent end-to-end coaptation and showed improvements in both corneal sensation and visual acuity. The loss of sensation in the anterolateral forearm was considered minimal and an acceptable outcome.

#### 4.4.2. Surgical Techniques of Fascicle Fixation

**Figure 7:**
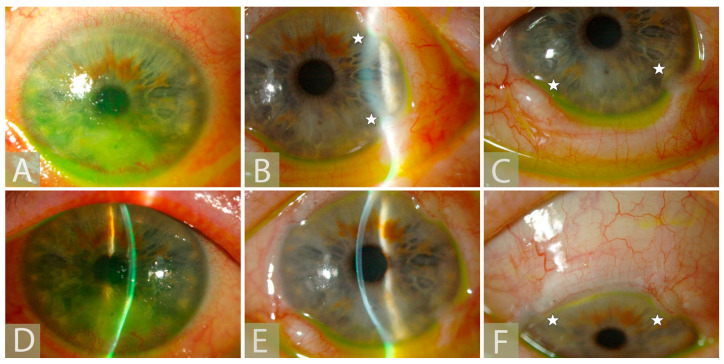
Slit lamp pictures of a 75 year-old patient of ours with NK Mackie stage III who has undergone ICN by ipsilateral GAN using sural nerve graft interposition: (**A**,**D**) Before corneal neurotization; (**B**,**C**,**E**,**F**) Three months after corneal neurotization (white stars show corneoscleral pockets).

Once the nerve fascicles are brought to the cornea, they can be fixated to the limbus in several ways. Some groups use fibrin glue and others simply suture the nerve fascicles to the limbus, while others leave them free in the sub-Tenon’s space (Figure 6B) [27,36,38,39,40,41,43,44,45,47,49,51,54,56,65,66,67,68]. Subsequent reports have adapted the technique by introducing a novel way of securing the peripheral fascicles into corneoscleral tunnel incisions. This is also our department’s preferred technique (Figure 6C,E,F; Figure 7; Video S1), as well as that of many other surgeons [28,52,69]. While this approach of placing the fascicles directly into the corneal tunnels may increase the axons available to reinnervate the cornea, it may reduce the possibility of restoring sensation to the bulbar conjunctiva that can occur with paralimbal fixation. In order to address this problem, Malhotra et al. use a combination of corneoscleral tunnel fixation and one or two supplementary fascicles that are left in the perilimbal sub-Tenon’s space and secured with fibrin glue within the area of the palpebral aperture (Figure 6D) [28]. The clinical relevance of conjunctival innervation has not been fully explored. Catapano et al. compared cases where fascicles were placed in the perilimbal subconjunctival space with those where the fascicles were secured into corneoscleral tunnels [50]. The latter showed faster recovery of central corneal sensation, usually in the first 3 months post operatively, but there were no more differences between the two groups at 6 months postoperatively.

#### 4.4.3. Postoperative Complications and Donor Site Morbidity

The sural nerve is the most frequently harvested autogenous nerve graft for ICN, as well as general peripheral nerve reconstruction. For the latter, other commonly used autografts include medial and lateral ABCN, the superficial branch of the radial nerve and the dorsal cutaneous branch of the ulnar nerve. A recent literature review analyzed chronic postoperative complications and donor site morbidity after sural nerve autograft harvest. The meta-analysis found that 22.9% of patients may have chronic pain, 7% may have wound complications, and 7.9% may have symptoms that noticeably affect their daily life. The authors suggest that considering these complications, alternative mediums for nerve reconstruction should be explored [70]. Rafailov et al. showed in a retrospective study high patient satisfaction and minimal postoperative pain and morbidity [71]. Catapano et al. looked at sensory and functional morbidity following sural nerve harvest in pediatric patients (14 cases). They found that despite objective loss of innervation, most patients have minimal morbidity, with half of the patients reporting no subjective evaluation of sensory loss. Nevertheless, a quarter of the patients had occasional pain, dysesthesia, or cold hypersensitivity and one patient reported inability to perform activities due to the pain sensation in his foot [72].

### 4.5. Indirect Corneal Neurotization—Acellular Nerve Allograft

**Figure 8:**
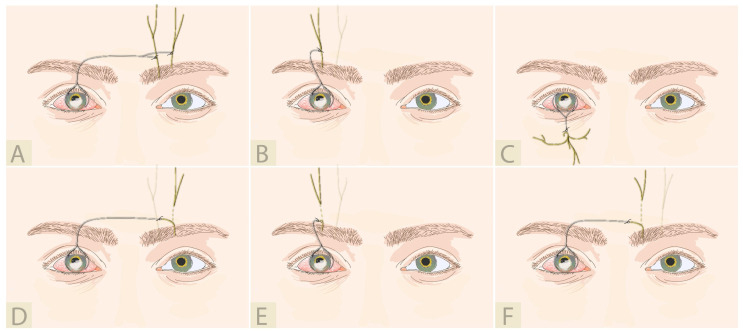
Indirect corneal neurotization using nerve allograft: (**A**) End-to-side allograft to contralateral ST and SO [67]; (**B**) End-to-side allograft to ipsilateral SO [67]; (**C**) End-to-side allograft to ipsilateral IO [67,73]; (**D**) End-to-end allograft to contralateral SO [67,73]; (**E**) End-to-end allograft to ipsilateral SO [67,73]; (**F**) End-to-end allograft to contralateral ST [73].

While indirect techniques offer a number of advantages over direct neurotization, harvesting of the nerve graft extends the surgical time and increases the risk of possible morbidity. Substituting the autologous nerve graft for an “off-the-shelf” product therefore offers a potentially safer alternative. Acellular nerve allografts (ANA) have been used to surgically repair the many causes of peripheral nerve injury and, in 2019, Leyngold et al. reported the use of an off-the-shelf processed human nerve allograft for MICN [73] (Figure 8). A 7 cm processed acellular nerve allograft was used successfully for ICN in seven patients. All patients showed improvement in corneal sensibility, shown both by Cochet-Bonnet esthesiometry and in vivo confocal microscopy. While the use of ANA offers several advantages, there could be some potential downsides. In addition to the costs associated with purchasing an ANA product, issues regarding storage and limitation of the graft length (7 cm) have led some researchers to state that ANAs are an inappropriate graft choice for corneal neurotization [74]. Being acellular, ANAs lack the Schwann cells that play an important role in axon regeneration and there is little evidence for their efficacy in the repair of long nerve defects. Moreover, Jowett et al. hypothesized that a graft of 7 cm might not be long enough to offer a tension-free neurorrhaphy [74].

Subsequent clinical data reported from a retrospective multicenter case series using ANAs for ICN suggest that it is a safe and effective procedure [67]. At the last available follow-up, there were no complications reported for the 17 cases. An increase of corneal sensation was detected at a mean of 3.7 months postoperatively and visual acuity remained unchanged. There were no statistical differences in outcomes based on type of coaptation (end-to-end or end-to-side), donor nerve selection (supraorbital or infraorbital), or laterality of donor nerve (ipsilateral or contralateral), although the cohort itself was too small to draw definitive conclusions.

### 4.6. Direct vs. Indirect Technique Comparisons

While the literature shows that both DCN and ICN are safe and effective in restoring corneal sensation, comparative data are sparse. A multicenter prospective comparative study of 26 cases showed that the mean time to clinical improvement of the NK was 3.9 months and did not differ between the two techniques [69]. One year postoperatively, the outcomes were also comparable. The comparative analysis suggested that DCN might achieve faster clinical resolution in the early postoperative period (3 to 6 months) when compared to ICN, though the trial was not constructed as a randomized controlled study and there was considerable clinical variation between the two groups. A meta-analysis of outcomes including 17 studies showed that both techniques are likely to yield similar levels of benefit [75].

## 5. Cadaveric Studies Underlying Corneal Neurotization

The ideal candidate nerve to be used to innervate the cornea should be surgically accessible, robust, resistant to manipulation, high in axonal loads and preferably only innervating a relatively small or non-critical sensory cutaneous region which would be lost in the procedure. Cadaveric studies have greatly assisted the optimization of the various neurotization approaches and have improved the efficacy of this relatively new approach in patients.

Domoshek et al. compared the distal supraorbital to the supratrochlear nerve, indicating that the former contains approximately 1000 more axons than the latter (3146 ± 1069 versus 1882 ± 903), which is an important consideration in direct reinnervation techniques [76]. If the supraorbital nerve is approached very proximally, just above the orbital rim, it contains yet more axons, suggesting that connecting the nerve (or coaptation) to a nerve graft should be done as proximal as possible to maximize the axonal load [77]. The same study by Domoshek et al. reported that the sural nerve, harvested as a conduit graft, contained 3176 ± 1524.5 myelinated nerve fibres. The number of nerves that reach the cornea via the sural nerve in these cases is more likely to be determined by the nerve to which it is surgically attached, rather than the number of fibres that are present in the sural graft conduit.

Despite their differences, both the supraorbital and supratrochlear nerves can be reliably dissected and used for neurotization. An anatomical study on 10 cadavers performed by Kikura et al. showed that both nerves have sufficient length for ipsilateral transfer and could be rotated to the center of the orbit without damage induced by excessive tension, supporting their use for neurotization [78]. Similarly, Leyngold et al. showed how a minimally invasive endoscopic technique could be used to perform an ipsilateral supraorbital nerve transfer while avoiding the complications of an open coronal approach [41]. The GAN and the infraorbital nerve have also been used as donor innervators, albeit to a much lesser extent, though the GAN is considered a good option for other nerve grafting repair procedures [79]. Altafulla et al. reported that the mean length of the nerve was 74.86 mm, and the mean diameter of its distal, middle, and proximal portions was 1.51, 1.38, and 1.58 mm, respectively [79]. A similar study including a larger number of cadavers found a similar average GAN length of 87.61 mm but with a larger mean width from all dissections. The nerve density of the GAN also makes it a promising candidate, with a mean number of myelinated axons per nerve of 6530 (ranging from 3756 to 9464) [80]. The infraorbital nerve has been studied to a far less significant degree. Catapano et al. found that the infraorbital nerve used to contain 975 myelinated fibres in an anatomic and histomorphometric feasibility study [81]. The authors commented that this number is expected to provide a sufficient number of donor axons to protect the sural nerve graft.

## 6. Clinical Measurement and Outcome Reporting in Neurotization

While the surgical procedure is slowly gaining in popularity, we are still missing uniformity in reporting outcomes to validate the reinnervation process. Until now, the outcomes mostly relate to the restoration of the cornea sensation and epithelial integrity but lack direct analysis of the trophic support for the ocular surface. In this section, we review the methodologies used to date, as well as issues to consider for future studies.

### 6.1. Corneal Esthesiometry

Esthesiometry is the simplest means of testing the corneal nerve function directly [82]. In 1894, Maximillian von Frey first proposed the concept of quantifying changes in corneal sensitivity by using different calibers and lengths of hair filaments [83,84,85]. In routine clinical practice, corneal sensitivity is often measured by teasing out the tip of a cotton bud to create a long, soft filament and using it to gently touch the cornea. While this approach is adequate for a general qualitative assessment of corneal sensation, it is not sufficient for clinical studies in which quantitative data are preferable. The most commonly used device for basic quantitative measurements of corneal response to mechanical stimuli is the Cochet Bonnet Contact Esthesiometer (CBA). The CBA uses a fine nylon filament to test the surface of the cornea in specific regions. The shorter the filament (in cm), the more pressure is applied, so an improvement in corneal sensitivity is represented by an increase in length of filament that can be felt at threshold. The CBA is widely used to report outcomes of corneal neurotization, not in small part due to its portability and ease of use. Nevertheless, there are some key limitations to this method, such as lower reproducibility related to user-dependency and little accuracy at a low threshold of stimulus with a tendency for underestimation even in normal subjects [86]. Moreover, the results obtained apply only to mechanoreceptors and the CBA has a limit of the minimum intensity stimulus possible, related to its 6 cm thread length [87].

The Belmonte non-contact corneal esthesiometer and its modified version (CRCERT-Belmonte) solve a number of these problems, but they are costly, difficult to obtain (as the previously available commercial product is no longer on the market), and may have to be custom built [88,89]. These pneumatic devices provide a wider range of mechanical, chemical, and thermal stimulus intensities all the way down to zero intensity, and are capable of accurate and repeatable corneal sensitivity assessment [87]. The non-contact esthesiometer is also able to measure corneal sensitivity at thresholds below the limits of the CBA. The results of CBA and non-contact corneal esthesiometer do not correlate, however, and cannot be compared directly [82,87]. The CBA may only represent mechanical pressure while the non-contact esthesiometer is more likely to represent the response to temperature change, as the air-pulse stimulus has been shown to possess a significant thermal element [86,88,90,91].

The CBA is still the most practical choice for functional testing after neurotization [92] given its wide availability, cost, and ease of use, although the limitations should still be borne in mind. The lower level of reproducibility is particularly relevant when considering the timing of post-neurotization sensitivity measurements. Given that the minimum level of intensity stimulus is relatively high, it is possible that early and lower-than-threshold sensorial function may escape detection. Since it also relies on patient reporting, the sensation “felt” by the neurotized cornea may be mapped to the previous dermatome and the patient may not necessarily interpret it as a positive result in the early stages. This may explain some of the conflicting evidence found in the literature where some patients report no corneal sensitivity at 12 months, despite evidence of corneal nerves on confocal microscopy [69].

### 6.2. Corneal Nerve Imaging by Confocal Microscopy

In vivo confocal microscopy (IVCM) is a useful clinical diagnostic tool that can offer information about corneal anatomy at a cellular level [93]. In the context of corneal nerves, its utility ranges from analyzing the sole presence or absence of the nerves, to assessing nerve morphology and pathological alterations, as well as trophic-related corneal changes. IVCM has become one of the more often-reported outcome measure in corneal neurotization both in establishing the diagnosis of NK and in the validation of the reinnervation efforts [50,51]. The mean nerve fibre density decreases with the severity of NK and can progress to the point where there is a complete absence of detectable nerves on IVCM [13]. After surgical neurotization, the exact evolution from perilimbal or intrastromal pocket donor nerve fixation to formation of a sub-basal layer is not completely known. Several studies have found microscopic evidence of nerve regrowth in IVCM as early as 3 months postoperatively in both DCN and ICN, with one case report already showing the reappearance of the sub-basal neural plexus at 8 weeks postoperatively in a young patient (14-years old) [55].

**Figure 9:**
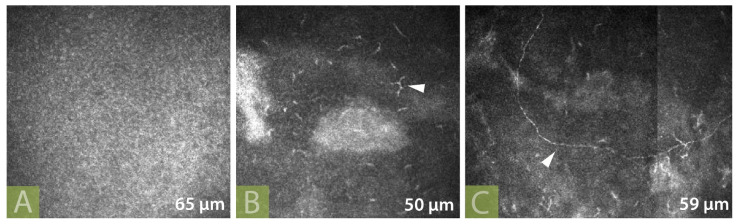
In vivo confocal microscopy of a 75 year-old patient of ours with NK Mackie stage III who has undergone ICN by ipsilateral GAN using sural nerve graft interposition: (**A**) Preoperatively; (**B**) At 3 months after corneal neurotization (white arrow shows sprouting neurons); (**C**) At 6 months after corneal neurotization (white arrow shows a nerve at the level of the sub-basal layer).

Nevertheless, it seems that the regenerated nerves do not always follow the normal anatomical pattern in the early postoperative phase [40,45,65,82]. Jowett et al. showed axonal sprouting within the sub-basal layer 3 months after ICN and obvious nerve imaging at 8 months postoperatively [61]. We noticed sprouting neurons during the 3-month follow-up and nerves at the sub-basal layer (59μm) at 6 months after ICN in one of our patients (Figure 9). Fung and Catapano described similar nerve sprouts in a more complex case in which a DALK was performed 45 months after ICN [51]. Fifteen months later, IVCM showed evidence of multiple branching short nerve stumps at the interface between the anterior stroma and the sub-basal layer.

### 6.3. Corneal Epithelial Morphology by Confocal Microscopy

Collateral signs of reinnervation, such as trophic-related improvements of corneal epithelium integrity and epithelial cell density, can also be evaluated by IVCM. Pathological changes in the corneal epithelium can manifest as increased proliferation, increased density of basal epithelial cells, and reduced superficial epithelial cell density, reflecting the higher loss of surface epithelium and epithelial defects seen in disease states [94,95]. Using IVCM, both DCN and ICN methods have shown objective improvements in corneal epithelial morphology [43,45,55]. In one case, Meniccaci et al. found that the density of the epithelial superficial cell layer increased over a follow-up period of 1.5 years, while the number of epithelial basal cells showed a progressive reduction which approached a more physiological phenotype and was ultimately comparable to the contralateral healthy eye [21].

### 6.4. Epithelium and Corneal Nerve Imaging by Anterior Segment OCT

Lathrop et al. assessed the clinical evolution of the epithelium of the corneal limbus in a case of ICN using optical coherence tomography (OCT) [53]. In the two-year follow-up after neurotization, this group observed a return to normal epithelial thickness over the cornea and limbus. This study suggested that the beneficial effects of neurotization could be mediated by improvements in the anatomy of the palisades of Vogt. Whether preferential positioning of the nerve fascicles at the 12 and 6 o’clock positions, where corneal limbal stem cells are most prevalent, would generate better results is yet to be determined.

Imaging of intrastromal corneal nerves using OCT, however, is only possible with spectrometer-based ultrahigh-resolution OCT (UHR-OCT) [68]. OCT is non-contact and much easier to use when compared to confocal microscopy, but there are some limitations. When evaluating the outcomes of corneal neurotization, UHR-OCT images mostly intrastromal nerves and is limited in its evaluation of the sub-basal nerve plexus. IVCM still provides superior quantitative and qualitative information about the corneal nerves and epithelium.

### 6.5. Lacrimal Gland Secretion

The majority of the sensory nerve fibres innervating the cornea are polymodal, and the reflex tear secretion caused by corneal stimulation appears to be mediated by these polymodal nociceptors [11,96]. Selective excitation of mechanoreceptors or cold receptors is less effective in releasing an enhanced lacrimal secretion. The afferent limb of the lacrimal functional unit consists of corneal and conjunctival sensory nerves which stimulate the efferent parasympathetic and sympathetic innervation to the lacrimal, Meibomian, and associated glands [97]. NK has a suppressive effect on the nasal–lacrimal reflex and the resulting tear deficiency is another factor contributing to corneal epithelial breakdown in these cases [98]. Repairing corneal sensation, in the best-case scenario, should also re-establish and improve the diminished tear reflex. Tear measurements are, therefore, often included in the clinical assessment of corneal neurotization outcomes.

The Schirmer test is one of the most common diagnostic tools in the evaluation of tear production in clinical use. Schirmer I is performed without anesthesia and measures the basal tear secretion together with the conjunctival–lacrimal trigeminal reflex [99]. Measurement of the basal tears alone can be performed similarly but with topical anesthesia (Schirmer II). Despite its low degree of reproducibility and reliability, Schirmer testing remains popular as a quick and inexpensive way to measure tears, but the high variability makes it a less-suitable choice for corneal neurotization follow-up.

Bourcier et al. reported the outcomes of the Schirmer I test after corneal neurotization and no difference in tear production pre- and postoperatively, nor was there a difference between the operated and contralateral eye [59]. This result is understandable because while the afferent inputs of the lacrimal functional unit (LFU) can be attributed to a single eye, the central nervous system integration of the reflex connects the afferent limb to the efferent arms bilaterally, thereby causing tearing in both eyes. There may also be asymmetry between the production capacity of the tear glands in these diseased eyes. This makes contralateral eye comparison per patient very difficult. Conversely, Giannacare et al. reported that the Schirmer test I improved from the mean preoperative values of 3.0 mm/5 min to 7.66 mm/5 min for three cases at 12 months postoperatively [39]. The mean value for the contralateral eye was 8.33 mm/5 min. This suggests that the Schirmer I test is influenced by too many variables to allow the contralateral eye to act as a control in these patients.

Another means of assessing the tear volume is by imaging the tear meniscus using anterior segment OCT [100]. This concave reservoir of tears is located at the lower and upper lid margin and represents the basal volume of tears. This approach is limited by the same issues as those of the Schirmer test but also on the position of the eyelids. Many cases of corneal anesthesia treated by corneal neurotization stem from mixed nerve palsies or post-surgical transections. In these cases, the facial nerve is often involved and results in laxity of the lower eyelid, thereby making the accurate measurement of a tear meniscus near impossible.

### 6.6. Blink Reflex

Terzis et al. reported the postoperative evaluation of the blinking reflex; however, this was subjectively assessed in a psychosocial follow-up questionnaire [36]. Three patients evaluated their own blink reflexes on a scale of 1–10 and reported a range of 1.5–8. The average time from the date of surgery to completion of the questionnaire was 16.3 ± 2.42 years, making these data difficult to interpret [36].

Neurophysiological studies aiming to measure the blink more objectively have been performed after DCN [39]. This study involved placing a cathode to the peripheral temporal cornea and an anode on the temporal orbital region in the area of the orbicularis oculi muscle. The eyes were then stimulated using an electrical impulse of 0.2 milliseconds, and the intensity adjusted for each patient, based on the healthy eye’s sensory threshold. Three patients who underwent DCN were included, and were tested preoperatively, and at 3, 6, and 12-months after surgery. The reflex was absent preoperatively and was partially recovered in all cases within 6 months after surgery [39]. In a larger study from the same group, 26 eyes from 25 patients who underwent either ICN or DCN were examined using this neurophysiological approach [69]. Partial recovery of the electrical response was seen at the 1-year postoperative visit, though this was not found to be correlated to the corneal sensitivity and corneal reflex parameters.

### 6.7. Sensation of the Bulbar Conjunctiva

Similar to the cornea, the sensory innervation of the bulbar conjunctiva is derived from the ophthalmic branch of the trigeminal nerve. Malhotra et al. have adapted their surgical technique to address the problem of an anesthetic conjunctiva in NK (Figure 6D) [28]. In addition to inserting four-to-five nerve fascicles into the corneoscleral tunnels, they also prepare one or two extra fascicles specifically for the conjunctiva which are secured in the perilimbal sub-Tenon’s space at 3 and 9 o’clock using fibrin glue. The aim of this modification is to restore sensation to the bulbar conjunctiva in the region of the palpebral aperture. The authors also suggested that monitoring the sensation of the bulbar conjunctiva after corneal neurotization should be included in the outcome measures of future studies.

### 6.8. Cortical Remapping and Functional Neuroimaging

When sensory nerves are diverted from one dermatome to another region, recuperation of sensation involves linking the corneal surface to a new region of the somatosensory cortex.

Cortical plasticity may therefore play an important role in the recovery of conscious sensation of the cornea [65]. Several authors have reported a misperception of the corneal tactile stimulation in the cutaneous area supplied by the donor nerve in the early postoperative phase [61,73]. These abnormal sensations are related to the source of the donor nerve. Some patients feel ocular irrigation as a cold sensation in the earlobe when the GAN is used, or in the ipsilateral scalp when the supratrochlear nerve is used. These dissociations in perception usually resolve at around 6 months postoperatively.

In one reported case, the neurophysiological pathways involved in the corneal reinnervation were examined using magnetoencephalography (MEG) [50]. The patient suffered from NK in his right eye and underwent MICN using the left supratrochlear nerve as the donor nerve. Immediately postoperatively, MEG did not detect any sensory response during stimulation of the right neuropathic cornea. Eight months after MICN, an evoked response was localized in the right somatosensory cortex during mechanical right corneal stimulation. This area corresponds to the region that received sensation from the area supplied by the left supratrochlear nerve, confirming that the corneal response was elicited and felt via the donor’s left supratrochlear nerve [50]. While this process of cortical “re-learning” is not fully understood, Malhotra et al. proposed that some of the approaches to postoperative recovery applied in other fields, like hand surgery, could be of benefit post corneal neurotization [28]. Patients were able to enhance early sensory outcomes using early postoperative mirror visual feedback for the stimulation of sensory and motor cortex after median and ulnar nerve repair. At 6 months, discriminative touch was significantly better in the early intervention group. It could, therefore, be beneficial to use analogous exercises (such as using cold or warm eye drops or gentle corneal stimulation) directed at modulating cortical remapping with the goal of improving clinical outcomes after corneal neurotization [28].

## 7. Discussion

Both direct and indirect corneal neurotization seem to be feasible procedures with similar clinical results. To date, no specific surgical approach has come to the fore as clinically better, as even in the comparative studies, different surgical indications were considered. Most authors prefer the sural nerve as a conduit and the supraorbital and/or supratrochlear nerves as donors. When considering the direct or indirect approaches, it appears that both are commonly used, though there are more cases of the ICN in the published literature. The decision on which approach to use appears to be related more to the etiology of the NK, surgical site morbidity, age of the patient, experience of the surgeon, and the availability of the necessary equipment and interdisciplinary support.

The main goal of corneal neurotization is to restore corneal integrity and prevent possible devastating consequences of NK, such as persistent epithelial defects leading to melting and perforation. This is achieved by new nerve ingrowth from the donor nerve into the anesthetized cornea, which leads to different degrees of improvement of corneal sensibility and local homeostasis. Augmenting this treatment with NGF could improve outcomes, but we are not aware of any cases where this approach was tried to date.

There is no expected visual improvement by corneal neurotization alone, and patients should be well informed about this aspect. Nevertheless, by restoring corneal sensation, further surgeries for visual rehabilitation, such as DALK or PK, are made possible. The reported complications appear to be minimal and most of the post-operative problems occur at the surgical site of graft or donor harvest with paresthesia and transitory numbness being the most frequently described complications.

Most of the authors prefer to report their outcomes by corneal esthesiometry (CBA) and in vivo confocal microscopy. Even though these are very useful tools, they both have some downsides. The corneal esthesiometry can be unreliable and relatively subjective, while the IVCM is more accurate but also more invasive and cumbersome. Consensus and consistency in outcome reporting would greatly improve our ability to compare the efficacy of different surgical approaches.

## 8. Conclusions

Corneal neurotization is a relatively new, but promising, surgical approach for the treatment of moderate-to-severe neurotrophic keratopathy. It can restore corneal sensation by transferring healthy nerves to the anesthetic cornea, either directly or via a conduit, but it usually does not have a direct impact on visual acuity. The procedure is interdisciplinary and, while it is a relatively complex surgery, complications are rare.

The reported results suggest a high degree of success in recovering corneal sensibility and epithelial integrity. There is still no agreed standard in reporting outcomes and surgical follow-up, and to date the most frequently performed examinations are Cochet-Bonnet esthesiometry and in vivo confocal microscopy.

## Figures and Tables

**Table 1:** Corneal neurotization by indications reported in literature.

Etiology of Neurotrophic Keratopathy	Diagnosis
Neurosurgery	VIIIth CN ^1^ (acoustic) neuroma
	AVM ^2^
	Meningioma
	Trigeminal neuroma
	Condrosarcoma (pontocerebellar region)
	Trigeminal neuralgia
	Gamma knife surgery and microvascular decompression of the trigeminal nerve
	Acute hemorrhage of a pontine cavernoma
	Microvascular decompression of trigeminal nerve
	Unspecified
Viral	Herpes Simplex virus
	Herpes zoster virus
	Herpes Simplex virus or Varicella zoster virus
Trauma	
Ophthalmic surgery	Long ciliary nerves damage after retinal detachment surgery
	After ptosis surgery due to congenital oculomotor nerve palsy
Congenital	Cerebellar hypoplasia with bilateral corneal anesthesia
	Peripheral nerve dysfunction
	Hypoplasia of trigeminal nerve
	Agenesis of trigeminal nerve
	Idiopathic
	Congenital—other neurologic associations
Vascular	Ischemia of left posterior inferior cerebellar and distal vertebral artery
Diabetes Mellitus Type 2	
After unspecified corneal infection	
Radiotherapy after subtotal removal of an acoustic neuroma	
Prostatic bone metastasis	
Unknown origin	

^1^ Cranial nerve, ^2^ Arteriovenous malformation.

## Data Availability

No new data was created.

## Supplementary Materials

The following supporting information can be downloaded at https://www.mdpi.com/article/10.3390/jcm12062214/s1, Video S1: Corneal neurotization by ipsilateral GAN using sural nerve graft interposition.
